# Editorial: Temporal Cognition: Its Development, Neurocognitive Basis, Relationships to Other Cognitive Domains, and Uniquely Human Aspects

**DOI:** 10.3389/fpsyg.2019.01865

**Published:** 2019-08-13

**Authors:** Patricia J. Brooks, Danielle DeNigris

**Affiliations:** ^1^College of Staten Island and the Graduate Center, CUNY, New York, NY, United States; ^2^Department of Psychology & Counseling, Fairleigh Dickinson University, Madison, NJ, United States

**Keywords:** temporal cognition, temporal perception, temporal reasoning, autism spectrum disorder, spatial-temporal relations, mental time travel, motor timing, prospection

Human lives are organized around time. As a species, we manifest an acute interest in its passage as exemplified by the clocks, calendars, and other instruments used to mark time with precision. From early childhood, we acquire linguistic and other mental capacities to simulate travel from the ever-changing present into the past or future. Our abilities to perceive, estimate, and keep track of time, collectively described as temporal cognition, rely on multiple forms of representation. Temporal cognition underlies the development of episodic and autobiographical memory, foresight, and planning, and forms the basis for building a stable self-concept.

Studies of temporal cognition often distinguish lower-level perceptual mechanisms and higher-order capacities reliant on language and other symbolic media (Nunez and Cooperrider, 2013). Hoerl and McCormack (2018) offer a dual-systems approach, differentiating temporal updating mechanisms for tracking duration, elapsed time, and sequential order of events from temporal reasoning abilities. Temporal reasoning uses explicit formats to mark specific times/positions of events and mental simulation to imagine alternate realities. Like other forms of reasoning, it often relies on heuristics and is subject to bias.

For this Research Topic, we invited contributors to address the myriad ways temporal cognition impacts human behavior and psychological functioning, its development over the lifespan, and its uniquely human aspects. The first two papers aimed to characterize low-level perceptual mechanisms that track durations, intervals, and other temporal features of stimuli. Zeng and Chen examined perception of the time interval between an action and its sensory feedback, and demonstrated the robustness of our ability to average interval durations across these two modalities. Such temporal judgments may play a key role in the perception-action feedback loops that underpin coordinated behavior. Szelag et al. explored temporal resolution and sequencing abilities of healthy elderly adults, estimating separately their thresholds for perceiving temporal order of auditory stimuli varying in location (*right ear-left ear* vs. *left ear-right ear*) or spectral characteristics (*high-low* vs. *low-high*). The distinct response distributions and learning trajectories observed across the two tasks suggest that strategic processing influences low-level temporal perception.

Shifting to higher-level temporal cognition, Zhang and Hudson examined the interrelatedness of temporal reasoning and language development, asking whether language is necessary for the formation of temporal concepts and not just for the expression of such concepts. The next two papers focused on children with autism, a population that exhibits deficits in temporal cognition (Boucher et al., 2007; Lind and Bowler, 2010). Anger et al. found beneficial effects of visual cues in eliciting past and future autobiographical details from autistic adolescents, who produced markedly fewer details than neurotypical controls when assessed via free recall. Overweg et al. compared autistic and neurotypical children's comprehension of temporal conjunctions *before* or *after*. Autistic children performed worse than controls, with variance explained by receptive vocabulary, nonverbal abilities, and performance on a theory of mind task in which they made inferences about a person's beliefs about another person. The authors concluded that weak perspective-taking skills may account in part for children's difficulties in comprehending temporal expressions.

Next, we explore cross-domain mappings between space and time, as evident in the use of spatial terms to represent temporal concepts (e.g., the past is *behind*, the future is *ahead*; an earlier event is *left* of a later event). Observations that people use spatial terms to talk about time more often than temporal terms to talk about space has been taken as support for Conceptual Metaphor Theory—that people rely on concrete, highly structured experiences as a source for metaphorically representing more abstract experiences, e.g., representing *time* as *money*, as a valuable commodity and limited resource (Lakoff and Johnson, 1980).

Two papers in this issue challenge the assumption that the mapping across spatial and temporal domains is inherently asymmetric. Kranjec et al. used a cross-domain contamination paradigm to compare the extent to which temporal information influences spatial judgments and vice versa. The authors found bi-directional effects that varied with task modality, and concluded that visual-spatial and auditory-temporal associations are privileged relative to other mappings. Similarly, in their review of 16 empirical studies of spatial-temporal relations, Loeffler et al. found that studies supporting the asymmetric hypothesis tended to use visual tasks across spatial and temporal domains, whereas studies supporting the symmetric hypothesis used auditory tasks for temporal representations, but visual tasks for spatial representations. Modality effects are further corroborated by studies of lower-level statistical learning of probabilistic sequences, where participants exhibit superior learning of temporal order when stimuli are presented in the auditory as opposed to visual or tactile modalities (Conway and Christiansen, 2005).

Three papers discuss methodological issues associated with mental time travel. Demiray et al. examined the temporal orientation of mental time travel assessed via electronically activated recordings (EARs) of snippets of naturally occurring speech. In contrast to signal-contingent experience sampling, where people respond to randomly timed signals, the EARs were collected unobtrusively. Participants showed a retrospective bias in conversational time travel, talking about their personal past more than twice as often as their personal future. Walsh and Busby Grant address coding challenges associated with experience sampling methods where participants' momentary thoughts are collected via text prompts. Human coders were more accurate than automated text coding algorithms in judging the temporal orientation of the recorded experiences. Accuracy was low (<80%) across conditions, indicating difficulties associated with coding ambiguous text for temporal perspective. The authors stress the importance of collecting temporal information from participants while sampling their experiences.

The claim that mental time travel is a uniquely human capacity (Suddendorf and Corballis, 2007) has led to innovative research on the capacities of non-human primates and avians to plan for the future (Bourjade et al., 2012; Clayton, 2015). Martin-Ordas and Atance tested adult humans on a decision-making task adapted from animal research, where participants had to choose which of two foods they would want in the future when one (a popsicle) would no longer be edible. Despite knowledge that popsicles melt, adults performed poorly in making future judgments, underscoring how difficult it is to envision how one will feel in the future and the biasing impact of the present (Gilbert and Wilson, 2007).

The final papers focus on implications of individual differences in temporal processing for health and well-being. Young et al. found motor timing deficits to be predictive of self-perceived efficacy to abstain from substance use among individuals in treatment for alcohol and/or cocaine use. Bulley and Irish review the role of prospective cognition in goal-directed behavior and decision-making, and highlight clinically relevant changes in prospection associated with psychiatric disorders including dementia, depression, anxiety, and addiction.

Understanding how humans represent lived and imagined experience in infinite variation requires a grasp of how the mind tracks change over time. As the variety of contributions to this Research Topic indicates, temporal cognition is multifaceted in its expression over the lifespan. As a field of inquiry, temporal cognition benefits from recent efforts to develop integrative theoretical frameworks relating higher- and lower-level processing mechanisms. Much remains to be understood about how outputs of temporal perceptual processes are redescribed into more explicit formats to support everyday judgment and decision-making.

## Author Contributions

All authors listed have made a substantial, direct and intellectual contribution to the work, and approved it for publication.

### Conflict of Interest Statement

The authors declare that the research was conducted in the absence of any commercial or financial relationships that could be construed as a potential conflict of interest.
